# Repbase Update, a database of repetitive elements in eukaryotic genomes

**DOI:** 10.1186/s13100-015-0041-9

**Published:** 2015-06-02

**Authors:** Weidong Bao, Kenji K. Kojima, Oleksiy Kohany

**Affiliations:** Genetic Information Research Institute, 5150 El Camino Real, Ste B-30, Los Altos, CA 94022 USA; Department of Computational Biology and Medical Sciences, Graduate School of Frontier Sciences, University of Tokyo, Minato-ku, Tokyo Japan; Institute of Medical Science, University of Tokyo, 4-6-1 Shirokanedai Minato-ku, Tokyo, 108-8639 Japan

**Keywords:** Repbase Update, Repbase Reports, Transposable element, RepbaseSubmitter, Database

## Abstract

Repbase Update (RU) is a database of representative repeat sequences in eukaryotic genomes. Since its first development as a database of human repetitive sequences in 1992, RU has been serving as a well-curated reference database fundamental for almost all eukaryotic genome sequence analyses. Here, we introduce recent updates of RU, focusing on technical issues concerning the submission and updating of Repbase entries and will give short examples of using RU data. RU sincerely invites a broader submission of repeat sequences from the research community.

**Electronic supplementary material:**

The online version of this article (doi:10.1186/s13100-015-0041-9) contains supplementary material, which is available to authorized users.

## Background

Repbase Update (RU), or simply “Repbase” for short, is a database of transposable elements (TEs) and other types of repeats in eukaryotic genomes [1]. Being a well-curated reference database, RU has been commonly used for eukaryotic genome sequence analyses and in studies concerning the evolution of TEs and their impact on genomes [2–6]. RU was initiated by the late Dr. Jerzy Jurka in the early 1990s and had been developed under his direction until 2014 [7]. Currently, RU continues to be maintained by the Genetic Information Research Institute (GIRI). Free access to RU data is registration-based for academic and non-profit researchers, but a licensing agreement is needed for commercial users. RU and other libraries derived from it are downloaded around 500 times a month from our web server (http://www.girinst.org). In the past 3 years, an average of 159 new users per month have been approved from around the world. As an e-journal accompanying the RU, “Repbase Reports” (RR, ISSN# 1534-830X) was launched in 2001 to better acknowledge the original contributors to RU entries and to serve as a permanent record. The availability of RU, its data format and implementation, and supplemental tools (Censor and RepbaseSubmitter) were detailed in 2005 and 2006 [1, 8]. This brief paper will focus on recent updates of RU, technical issues concerning the submission and updating of Repbase entries, and will give short examples of using RU data.

### RU and TE identification

In eukaryotic genomes, most TEs exist in families of variable sizes, i.e., TEs of one specific family are derived from a common ancestor through its major burst of multiplication in the evolutionary history. A consensus sequence can be reconstructed for each family to approximate the sequence of its ancestral active TEs. Consensus sequences were used to experimentally reconstruct active TEs for transgenesis and insertional mutagenesis [9]. Consensus sequences are especially valuable when classifying TEs and masking repeats, particularly for “old” families of which the sequences have been highly degenerated. The distance from each copy to the consensus is approximately half of the distance between two copies. Family age can be indicated by the average sequence divergence between the consensus and the family members [10].

**Fig. 1:**
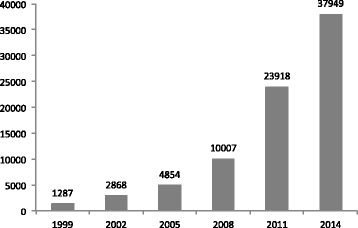
Numbers of the entries in Repbase Update since 1999

RU currently contains more than 38,000 sequences of different families or subfamilies, which almost doubled every 3 years since 1999 (Fig. 1). Over 70 % of these entries are complete consensus sequences, unreported elsewhere. The other 30 % of entries represent sample sequences extracted from individual loci (in some cases, the sequences are incomplete). Approximately 90 % of the RU families/subfamilies are collected from a total of 134 species (at least 50 TE families each, Table 1). The remaining 10 % are composed of repeats from another ~700 species. For the complete list of species and their entry numbers, see Additional file 1. RU also stores non-TE repeat sequences such as satellite sequences, microsatellites, multi-copied RNA genes (rRNA, tRNA, snRNA), and some integrated viral sequences—but these types of sequences are not as thoroughly collected as in other dedicated databases, such as SILVA ribosomal RNA gene database [11], 5S ribosomal RNA database [12], GtRNAdb [13], and “paleovirology” (http://bioinformatics.cvr.ac.uk/paleovirology/).

**Table 1:** Top 134 species account for 90 % of the entries

Taxonomic group		No. of species	No. of family
Chromalveolata	Alveolata	1	105
	Oomycetes	3	732
Opisthokonta	Fungi	8	1595
	Cnidaria	3	1465
	Echinodermata (*Strongylocentrotus purpuratus*)	1	282
	Hemichordata (*Saccoglossus kowalevskii*)	1	105
	Lophotrochozoa	3	1122
	Nematoda	2	411
	Arthropoda	35	8161
	Branchiostomidae (*Branchiostoma floridae*)	1	265
	Tunicata	2	276
	Vertebrata (non-mammalian)	22	9171
	Mammalia	28	4353
Chromalveolata	Rhodophyta (*Chondrus crispus*)	1	1181
	Chlorophyceae	2	220
	Embryophyta	29	8101

Exhaustive identification of TEs and the reconstruction of family consensus sequences in complete length are a time-consuming process. Moreover, the challenge of identification varies in different genomes regardless of their size. There are a number of tools designed to automate TE identification and/or annotation, such as RECON [14], RepeatScout [15], PILER [16], RepeatModeler [17], Dfam [18], REPCLASS [19], REPET [20], and PASTEC [21], with none having distinct advantages [20, 22]. It is noteworthy that these tools use RU as a reference in the classification and annotation process. At GIRI, TE identification mostly involves multiple rounds of running a homemade pipeline based on RECON [14]. A majority-rule consensus sequence is reconstructed from the multiple sequence alignment for each family, and the CpG doublets are optionally compensated for in the consensus, especially in mammalian repeats. About 10–20 sequences are usually sufficient to generate a decent consensus, but fewer sequences can also be used for families of smaller size. In most cases, consensus sequences are manually extended to their real termini, since terminal sequences and TSDs are critical to the classification of TEs (especially non-autonomous ones). For older families, the consensus is often constructed through a two-step process: a pre-build consensus is used to select top-hit sequences and then these sequences are used to build the consensus one more time. Another frequently used tool for TE identification is LTR_FINDER [23], which is used to detect LTR retrotransposons.

### TE annotation, classification and naming

In RU, TEs are currently classified into three groups, i.e., DNA transposons, LTR retrotransposons (including retrovirus), non-LTR retrotransposons (including the SINE category) [24], and further into 65 superfamilies or clades (*MuDr*, *hAT*, *SINE1*, *L1* etc.) (Table 2). Conventionally, the term “superfamily” refers to DNA transposons, while the term “clade” applies more frequently to non-LTR retrotransposons and LTR retrotransposons. The classification of autonomous DNA transposons is relatively straightforward, often performed by similarity searching of predicted coding sequences, such as by BLASTP or PSI-BLAST. In principal, transposases of one superfamily should not converge with proteins of another when using the PSI-BLAST (an e-value less than 0.005 would be considered converged after several rounds of iterations) [25, 26]. However, this criterion is overridden if two superfamilies converge separately with different groups of bacterial transposases, such as *Zator* and *Mariner* [26], or if peculiar features are found with certain remote groups, such as *Dada* and *MuDr* [27]. In addition, a superfamily may consist of several distinct subgroups, among which the divergence is insufficient for them to be viewed as distinct superfamilies, such as *Sola1*, *Sola2*, and *Sola3* in the *Sola* superfamily [26]. So far, the classification of LTR retrotransposons is also straightforward, but the classification of autonomous non-LTR retrotransposons is largely phylogeny-based instead. For their classification, GIRI provides an online service, called RTclass1 [28], at http://www.girinst.org/RTphylogeny/RTclass1/. Notably, the classification is subject to ongoing updating once new meaningful data or superfamilies/clades emerge. For example, three previous superfamilies have been recently reclassified into *EnSpm* (*Chapaev*, *Mirage*) and *MuDR* (*Rehavkus*) based on weak but significant sequence similarities [24, 29]. Meanwhile, a number of superfamilies were added in recent years—*Academ* [30], *Zisupton* [31], and *Dada* [27] to name but a few. The classification of non-autonomous DNA transposons is largely based on their terminal sequences, TSD features, TIRs, and other structural features (e.g., terminal hairpin in *Helitrons*). If two non-autonomous DNA TEs have the same TSDs in length and show terminal alignment from position 1 to 11 (1 mismatch allowed), they are annotated in the same superfamily in RU.

**Table 2:** Transposon classification in Repbase

Group	Superfamily/clade
DNA transposon	*Academ* ^a^, *Crypton* ^a^ *(CryptonA* ^a^ *, CryptonF* ^a^ *, CryptonI* ^a^ *, CryptonS* ^a^ *, CryptonV* ^a^ *)*, *Dada* ^a^, *EnSpm/CACTA*, *Ginger1* ^a^, *Ginger2* ^a^, *Harbinger*, *hAT*, *Helitron*, *IS3EU* ^a^, *ISL2EU*, *Kolobok*, *Mariner/Tc1*, *Merlin*, *MuDR*, *Novosib*, *P*, *piggyBac*, *Polinton*, *Sola* ^a^ *(Sola1* ^a^, *Sola2* ^a^, *Sola3* ^a^ *)*, *Transib*, *Zator* ^a^, *Zisupton* ^a^
LTR retrotransposon	*BEL*, *Copia*, *DIRS*, *Gypsy*, *ERV1*, *ERV2*, *ERV3*, *ERV4* ^a^, *Lentivirus* ^a^
Non-LTR retrotransposon	*Ambal* ^a^, *CR1*, *CRE*, *Crack*, *Daphne*, *Hero*, *I*, *Ingi*, *Jockey*, *Kiri* ^a^, *L1*, *L2*, *L2A*, *L2B*, *Loa*, *NeSL*, *Nimb*, *Outcast*, *Penelope*, *Proto1*, *Proto2*, *R1*, *R2*, *R4*, *RandI/Dualen*, *Rex1*, *RTE*, *RTETP*, *RTEX*, *Tad1*, *Tx1*, *Vingi* ^a^
	*SINE (SINE1/7SL, SINE2/tRNA, SINE3/5S, SINE4* ^a^ *, SINEU* ^a^ *)*

^a^Superfamilies/clades added since our latest classification reports [24, 28]

Each entry in RU, either consensus or sample sequence, represents a “family” or “subfamily” of TEs. Except for a small number of early submitted TEs, the entry name is formatted with the superfamily, subgroup, or clade name, followed by an Arabic number and the species abbreviation [24]. For example, *hAT-4_NV* and *hAT-4N1_NV* denote the autonomous family 4 of the *hAT* superfamily in *Nematostella vectensis* and the non-autonomous derivative family 1 of the former, respectively [24]. When non-autonomous TEs cannot be clearly classified with present knowledge, they are given general names, such as TE(DNA/LTR/non-LTR)-1_YY, where YY represents its host species. In RU the terms “family” and “subfamily” both correspond to the expanding events of TEs in one specific genome. “Subfamily”, however, connotates that two or more closely related TE families were derived from a common ancestral TE. Such subtle difference has more implications for the naming of TEs. Closely related subfamilies usually have similar names differentiated by short modifiers, such as the *AluSc* or *AluSq* subfamilies [10], or *CR1-3_LMi* and *CR1-3B_LMi* (see below). By contrast, different families are usually assigned with different Arabic numbers. The sequence similarities between retrotransposon families should be less than 80 % if both are consensuses, or less than 70 % if one is a sample sequence, over their whole length or shorter. If the two retrotransposons (consensus sequence or sample sequence) show greater than 80 % identity in more than 50 % of the shorter TE length compared, they are usually considered subfamilies of each other. For DNA transposons, especially *Helitron* or other long DNA transposons, the above criteria are still applicable in principle, but the similar regions are weighted favorably to their terminal sequences, rather than the other internal sequences, which could be accidentally captured alien sequences. One example of the subfamily naming convention is given by the retrotransposon sequences *CR1-3_LMi* and *CR1-3B_LMi*, which are 87 % identical to each other over their entire length. It should be mentioned that their naming does not mean that *CR1-3_LMi* is a family and *CR1-3B_LMi* is a subfamily; both are subfamilies if a common ancestral family is implied. To date, not all entries conform to this nomenclature, but they are subject to ongoing updating.

### RU updating

Records in RU are updated regularly. The date of the last update is recorded in each entry for tracking purposes. Updating occurs in different forms: substituting the original sample sequence with a consensus, refining or extending the sequence, adding protein sequences, removing alien (inserted or flanking) sequences, reclassifying, entry renaming, or deletion. The removed older entry versions can be found either in the appendix directory of our monthly RU release or in our archived RU releases (http://www.girinst.org/server/archive/). In part, updating is triggered when a batch of new sequences is to be incorporated into RU. Specifically, when the new sequences are compared to all existing sequences in RU, any pair of sequences showing sequence redundancy or name discrepancy will be reexamined. Additionally, some updating comes from candid suggestions by RU users. Credits for the contributors are added in the updated RU entry. To date, more than 5000 entries have been updated at least once.

### Submission to RU/RR

TE sequences can be submitted to the database, RU, or the e-journal, RR. All data published in RR will remain permanently archived and can be quoted like any other article published in a scientific journal. TEs and the accompanying commentaries published in RR are automatically stored in RU and distributed worldwide. Submitting sequences to RU or RR is highly encouraged. Doing so has the potential to increase the visibility of the research paper associated with the deposited sequences, and it should not interfere in any way with the publication of an associated analysis/description of the elements. Besides, the submitter can specify the release date of the submitted TEs by communicating this to GIRI. To date, only 2000 or fewer out of over 38,000 entries have been submitted by RU users outside of GIRI researchers.

Once editorially approved by the editors of RU/RR, the submitted sequences will be released. Any type of sequence, whether consensus or individual sample sequence, is acceptable, even if it is a fragment. However, complete consensus sequences with full annotation are preferred. The primary consideration for inclusion in RU is the novelty of the sequence, which can be checked conveniently at our Censor service (http://girinst.org/censor/index.php) [8]. In rare cases, if one sequence is nearly identical to any known sequence (for example, around 94 % identity or higher, over its whole length), it should meet one of the following conditions to be accepted to the database: (1) The sequences represent distinct subfamilies, showing at least one significantly divergent region (100-bp or more, insertion/deletion or less than 75 % identity). (2) Each subfamily presents in a large copy number, such as the many nearly identical *Alu* subfamilies. (3) The two sequences are identified in two remotely related species, where events of horizontal TE transfer are suggested. (4) The submitted sequence is of higher quality (showing intact ORFs, consensus vs. sample sequence) and is intended to replace the older one.

The submission procedure is performed through a Java-based interface called RepbaseSubmitter [8]. It is available for download at http://girinst.org/downloads/software/RepbaseSubmitter/.

The name (i.e., sequence ID in RU) of the submitted sequence is up to the submitter, but it should be simple and informative. By selecting the “Auto” tab on the “Summary” page during submission, RepbaseSubmitter will generate a unique name for the sequence based on its classification. The automatically generated name can then be modified to indicate whether the sequence represents a subfamily or a non-autonomous TE. In the “Reference” page of RepbaseSubmitter, users may be confused between the options to choose “Direct Submission to Repbase Update” or “Direct Submission to RR” in the “Submission” > “Select Repository” pull-down menu. The “RR” stands for “Repbase Reports”. If the sequence is a consensus and unreported elsewhere, RR is more suitable. Notably, RepbaseSubmitter does support batch submission by selecting the “Submit All” tab. However, this requires that all sequences be correctly IG-formatted, and each has a unique name. For this purpose, users can choose to save the RepbaseSubmitter-processed, properly formatted individual sequences into one file for the batch submission.

### Using RU

The monthly release of RU is available in both FASTA and EMBL formats. Only the EMBL files contain full annotations, such as TE classification, host species, release version, release date, latest update date, references, and comments. The EMBL files can be transformed into a relational database for local use. To detect repeat sequences in the genome sequences, FASTA-formatted RU data can be directly used with the standard homology search tools, such as BLAST programs, cross_match, and Censor [8]. Other TE annotation tools, such as RepeatMasker [32] and REPET [20], may need RU data in different formats. These tool-specific variants of RU can be downloaded from GIRI’s website, but they are prepared by the authors of the tools and are not updated on a monthly basis. The TE library used by RepeatMasker is essentially identical to the RU dataset, except for the format and the accompanying annotation-supportive files. However, it may show some minor sequence differences to RU at times for various reasons (see the README file in its package, downloadable at GIRI website http://girinst.org/server/RepBase/index.php). Various pre-masked genome sequences generated by RepeatMasker are available at the UCSC genome browser website (https://genome.ucsc.edu). In addition to RepeatMasker, RU is also essential for the Dfam database [18], where the profile hidden Markov models (profile HMMs) for different repeats are used in conjunction with the HMM search tool nhmmer to detect repetitive sequences in the genome [18]. Dfam is unique in that it does not rely on a homology-based search tools, but building profile HMMs is still dependent on the quality of the consensus sequences deposited in RU.

Depending on the specific aim, in many cases, only a subset of RU is needed. One can conveniently extract essential information by working in UNIX/Linux system. For example the AWK command, “awk 'BEGIN{FS = "//"; RS = "\0"; ORS = "//"}{for (i = 1; i <=NF; i++) if ($i ~ /\nKW.*hAT;/ && $i ~ / 7-bp TSD/) print $i}' XXX.ref”, will extract all those hAT families annotated with “7-bp TSD” from the EMBL file “XXX.ref”. With minor modifications, this command can also be used for extracting entries from a specific species or taxonomic group. Alternatively, on GIRI’s website, users can perform basic text searches at http://girinst.org/repbase/update/search.php, or search and download entries with specific taxonomic names or repeat classes at http://girinst.org/repbase/update/browse.php.

## Conclusions

For years, RU has been serving as a well-curated repeat library in virtually all eukaryotic genome research. At present, most entries in RU were submitted by researchers at GIRI and are not reported anywhere else besides RR. On the other hand, we highly encourage outside researchers to submit their repeat sequences to either RR or RU to expand the current repository of TEs thereby benefiting the whole research community. Meanwhile, RU will make every effort to keep up with the pace of newly sequenced genomes without sacrificing the established quality standards. Priority is placed on new genomes that are taxonomically less represented in RU. Suggestions for genomes to be analyzed are welcome. Until now, in each monthly release, RU entries are divided into several files according to the taxonomic origin. A number of entries may appear in multiple files (http://girinst.org/repbase/update/index.html). The separating of entries into individual files is becoming increasingly unnecessary, especially when the genomes analyzed are getting more diverse than before. For this reason, future releases of RU may be prepared as an all-in-one file, together with instructions and scripts to extracting the target groups. Another planned new feature of RU is a “Reference” protein library for each TE superfamily, which will comprise high-quality proteins only. This will be accomplished by using only recently active families. To avoid uncertainty derived from consensus building and/or exon-intron prediction, transposases will be selected from those without intron or with mRNA evidence. This protein set would be useful in understanding the diversity of TE-encoded proteins and their impact on the evolution of host genomes.

## Availability and requirements

- Project name: Repbase Update
- Project home page: http://www.girinst.org/repbase/update/index.html
- Operating system(s): Any system
- Programming language: N/A
- Other requirements: N/A
- License: a custom user agreement for RU
- Any restrictions to use by non-academics: license needed

## Additional file

Additional file 1:
**Species and the number of TE family in RU.** Data is up to the end of 2014.

## Abbreviations

GIRI: Genetic Information Research Institute
LTR: long terminal repeat
ORF: open reading frame
RU: Repbase Update
RR: Repbase Reports
TE: transposable element
TIR: terminal inverted repeat
TSD: target site duplication
